# Serum Thyroid-Stimulating Hormone and Anti-Thyroglobulin Antibody Are Independently Associated with Lesions in Spinal Cord in Central Nervous System Demyelinating Diseases

**DOI:** 10.1371/journal.pone.0100672

**Published:** 2014-08-05

**Authors:** Youming Long, Yangbo Zheng, Mengyu Chen, Bin Zhang, Cong Gao, Fulan Shan, Ning Yang, Yongxiang Fan

**Affiliations:** 1 Key Laboratory of Neurogenetics and Channelopathies of Guangdong Province and The Ministry of Education of China, Institute of Neuroscience and the Second Affiliated Hospital of GuangZhou Medical University, GuangZhou, Guangdong Province, China; 2 Department of Neurology, the Second Affiliated Hospital of GuangZhou Medical University, GuangZhou, Guangdong Province, China; 3 Department of Neurology, the Fifth Affiliated Hospital of GuangZhou Medical University, GuangZhou, Guangdong Province, China; University of Jaén, Spain

## Abstract

Transverse myelitis (TM) is associated with neuromyelitis optica (NMO) and multiple sclerosis (MS). Early recognition of useful parameters may be helpful to distinguish their difference. This retrospective study analyzed thyroid parameters from 243 serum samples (relapse = 128; remission = 115) of 178 patients with demyelinating diseases (NMO, n = 25; TM, n = 48; MS, n = 105). The relationship between thyroid and clinical parameters was analyzed. Patients with NMO and TM had a higher frequency of abnormal thyroid-stimulating hormone (TSH), anti-thyroglobulin antibodies (TG-Ab), and antithyroid peroxidase antibody (TPO-Ab) than MS patients (p<0.05). The level of TSH and TG-Ab returned to normal levels after administration of high-dose intravenous methylprednisolone (p<0.05). In 96 patients (NMO, n = 19; TM, n = 25; MS, n = 52) without treatment, serum levels of TSH, TG-Ab and TPO-Ab were significantly different between patients with and without myelitis (p<0.01). Patients positive for aquaporin-4 (AQP4) antibodies showed higher abnormalities of TSH (p = 0.001), TG-Ab (p = 0.004) and TPO-Ab (p<0.0001) levels than AQP4 antibodies negative patients. Logistic regression analyses revealed independent relationships between TSH (odds ratio [OR]  = 33.994; p<0.0001), TG-Ab (OR = 7.703; p = 0.017) and myelitis occurrence in 96 patients at the active stage. In 52 MS patients experiencing their first attack, MS patients with myelitis were associated with TSH abnormalities (OR = 42.778; p<0.0001). This study showed increased abnormalities of thyroid parameters in patients with NMO and TM than in MS patients. MS patients with myelitis also had greater TSH abnormality than in MS patients without myelitis. Abnormal TSH and TG-Ab were independently associated with myelitis occurrence in central nervous system demyelinating disorders.

## Introduction

Transverse myelitis (TM) is an inflammatory demyelinating disorder of the spinal cord that has various manifestations [1]. TM has several subtypes according to origin but in China the most common are neuromyelitis optica (NMO) spectrum and multiple sclerosis (MS) [1].

NMO is a severe, idiopathic, immune-mediated inflammatory, demyelinating and necrotizing disease characterized by transverse myelopathy and optic neuropathy. MS is also a chronic demyelinating disease whose lesions disseminate throughout multiple areas in the central nervous system (CNS), including the spinal cord and optic nerves. MS and NMO are considered distinct entities [2].

Recently, the identification of aquaporin-4 (AQP4) antibody as a diagnostic criterion [3] for NMO has facilitated its distinction from MS. However, details of the pathogenesis of MS and NMO are unknown, and many cases selectively involve the spinal cord and optic nerve. Early recognition of useful parameters may be helpful to distinguish the manifestations of MS, NMO and pure TM.

Autoimmune thyroid disease is a frequently studied disorder in MS [4], [5], [6], [7], [8], [9]. Most studies have focused primarily on the increased prevalence of thyroid dysfunction and antithyroid antibodies (ATAs) in MS patients compared with a control population. However, whether the frequency of thyroid disease in individuals with MS and their families is increased is controversial [10], [11]. Conversely, it is well known that NMO patients have increased levels of autoantibodies than MS patients [12]. Although thyroid diseases in the NMO spectrum in the Asian population has been described [13], [14], especially high-titer ATAs in patients with myelitis [5], [14], [15], [16], the significance of thyroid parameters in such demyelinating diseases is unclear.

The aim of this study was to evaluate whether there are differences in the abnormalities of thyroid parameters among subjects with NMO, MS or pure TM.

## Patients and Methods

The study protocol was approved by the Ethics Committee of the Second Affiliated Hospital of Guangzhou Medical University. Written informed consent was provided by all participants.

### Patients

A total of 354 Chinese Han subjects with CNS demyelinating disorders (between January 2008 – December 2012) were reassessed. Patients with NMO and MS were reassessed according to previously described criteria [3], [17]. In the present study, pure TM was defined as a patient characterized clinically by acute or subacute developing symptoms and signs of neurologic dysfunction in motor, sensory, autonomic nerves and nerve tracts of the spinal cord [18], but who did not meet the criteria of NMO or MS [3], [17].

Finally, 178 patients with available data were included in this study. None of the patients had known thyroid disease, or had a history of interferon treatment.

We analyzed thyroid parameters of 243 serum samples (relapse = 128; remission = 115) from 178 patients with demyelinating disease. MS patients comprised 64 females and 41 males with a mean age of 37.87±13.7 (12–74) years and 23.8% (25/105) had spinal-cord lesions according to MRI. Seventy-eight patients had two or more relapses, and 27 patients experienced their first attack. All NMO patients were females with a mean age of 44.6±15.95 (17–76) years and 88% (22/25) had two or more relapses. All NMO patients had spinal-cord lesions according to MRI. TM patients included 39 subjects with longitudinally extensive transverse myelitis (LETM; females/males = 30/9) and 9 patients with acute partial transverse myelitis (APTM; females/males = 6/3), with spinal-cord lesions confirmed by MRI [19]. The mean age was 45.92±13.23 (14–71) years and 47.9% (23/48) had two or more relapses. Nineteen TM patients were positive for AQP4 antibody and were considered having NMO spectrum disorders (NMOSD) [20]. Age, sex and spinal-cord lesions were significantly different between MS and NMO groups (Table 1). TM patients with AQP4 antibody were older (50.7±11.2 vs 42.8±13.3 years, p = 0.042), had a higher female/male ratio (18/1 vs 18/11, p = 0.001), and more relapses (73.7% vs 31%, p = 0.04) than TM patients without AQP4 antibody.

**Table 1:** Clinical characteristic of patients with NMO, TM and MS.

Characteristic	NMO (*n* = 25)	TM (*n* = 48)	MS (*n* = 105)	p*
F/M	25/0	36/12	64/41	<0.0001
Mean age**, y (range)	44.6±15.95 (17–76)	45.92±13.23 (14–71)	37.87±13.7 (12–74)	0.035
Relapses, n (%)	22/25 (88%)	23/48 (47.9%)	78/105 (74.3%)	0.144
Spinal lesions, n (%)	25/25(100%)	48/48(100%)	25/105(23.8%)	<0.0001

F, female; M, male; NMO, neuromyelitis optica; TM, transverse myelitis; MS, multiple sclerosis; n, number; y, year.

*Compared between NMO and MS.

**Mean value ± SD.

Data acquired from each admission record included age, sex, medication, number of demyelinating events, clinical characteristics, and the Expanded Disability Status Scale (EDSS) score [21] assessed at relapse and remission.

### Analyses of thyroid parameters in serum

Blood for thyroid parameters analyses was collected in the morning. For assessment of thyroid parameters, levels of free thyroxine (FT4), free triiodothyronine (FT3), thyroid-stimulating hormone (TSH), anti-thyroglobulin antibodies (TG-Ab), and antithyroid peroxidase antibody (TPO-Ab) were evaluated in serum samples by highly sensitive chemiluminescence immunoassay following the manufacturer's instructions. The normal range of the assays is 9–24 pmol/L and 2.5–6.5 pmol/L for FT4 and FT3, respectively. The normal range of TSH is 0.4–5.0 mIU/L. The normal range of TG-Ab and TPO-Ab is 0–50 KIU/L and 0–35 KIU/L, respectively. Thyroid parameters outside the normal range were defined as abnormalities, including low (<0.4 mIU/L) and high TSH (>5.0 mIU/L) levels.

### Immunofluorescence

AQP4-transfected cells (Euroimmun, Luebeck, Germany) and monkey thyroid tissues were obtained from a commercial kit (Euroimmun, Luebeck, Germany) [22]. After incubation with primary antibodies for 30 min (cell lines) or 2 h (tissues), the substrates were washed three times in PBS and incubated with anti–human or anti–rabbit IgG for 30 min, washed in PBS. Images were captured using a Leica microscope.

### Statistical analyses

All statistical analyses were conducted using the Statistical Program for Social Sciences ver11.0 (SPSS, Chicago, IL, USA). Statistical analyses were undertaken with the χ^2^-test or Fisher exact test for binary and categorical data. One-Way ANOVA and the Mann–Whitney *U*-test were employed for continuous variables. Risk factors for myelitis were analyzed by binary logistic regression analyses. Odds ratios (OR) and 95% confidence intervals (CI) for variables were calculated to estimate the impact on the occurrence of myelitis. p<0.05 was considered significant.

## Results

### Distribution of serum abnormal thyroid parameters in 243 samples

The Table 2 shows serum thyroid parameters in patients with different disorders.

**Table 2:** Distribution of serum abnormal thyroid parameters in 243 samples.

Diagnoses	Samples(n)	TSH (%)	FT3 (%)	FT4(%)	TG-Ab(%)	TPO-Ab(%)
Multiple sclerosis	126	17(13.5)	7(5.6)	1(0.8)	6(4.8)	8(6.3)
Acute exacerbation	60	16(26.7)	6(10)	1(1.7)	4(6.7)	7(11.7)
Remission	66	1(1.5)	1(1.5)	0	2(3)	1(1.5)
Neuromyelitis optica	49	24(49)	10(20.4)	2(4.1)	19(38.8)	24(49)
Acute exacerbation	30	20(66.7)	6(20)	1(3.3)	15(50)	20(66.7)
Remission	19	4(21.1)	4(21.1)	1(5.3)	4(21.1)	4(21.1)
Transverse myelitis	68	23(33.8)	16(23.5)	4(5.9)	20(29.4)	28(41.2)
Acute exacerbation	38	22(57.9)	11(28.9)	3(7.9)	15(39.5)	21(55.3)
Remission	30	1(3.3)	5(16.7)	1(3.3)	5(16.7)	7(23.3)

FT4, free thyroxine; FT3, free triiodothyronine; TSH, thyroid-stimulating hormone; TG-Ab, anti-thyroglobulin antibodies; TPO-Ab, antithyroid peroxidase antibody.

### Serum levels of TSH and TG-Ab return to normal after administration of high-dose intravenous methylprednisolone (ivMP)

**Figure 1:**
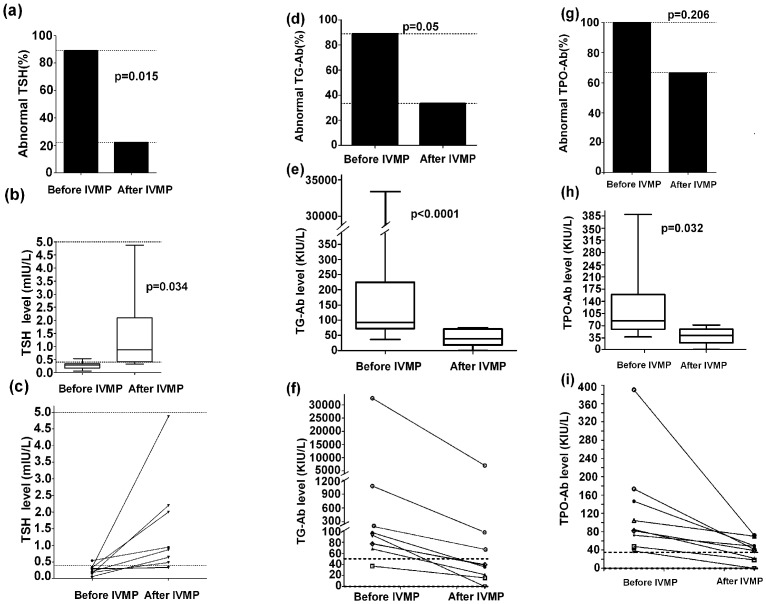
Changes in levels of TSH, TG-Ab, and TPO-Ab after ivMP administration in nine paired patients (a, b, c): changes of TSH; (d, e, f): changes of TG-Ab; (g, h, i): changes of TPO-Ab.

To ascertain whether all patients (treatment and without treatment) could be included in the further analysis, we must consider that therapy, such as ivMP, may affect thyroid parameters. Therefore, we prospectively observed thyroid function before and after one or two courses of ivMP in nine cases (NMO = 4, LETM = 5) who had not received therapy before ivMP. Paired sera were obtained shortly before ivMP and approximately 1 month after ivMP. Serum TSH increased (p = 0.034) and most cases returned to normal levels (p = 0.015) after ivMP. Conversely, serum TG-Ab (p<0.0001) and TPO-Ab (p = 0.032) decreased and returned to normal levels after ivMP (p = 0.05 and p = 0.206, respectively) (Table 3 and Fig 1a–i).

**Table 3:** Thyroid parameters of nine paired patients before and after IVMP.

Characteristics	Before IVMP	After IVMP	p
F/M	7/2	-	-
Mean age, y (range)	41.4±16.1 (20–68)	-	-
EDSS (mean value ± SD)	6.56±1.58	2.94±1.29	<0.0001
FT3 (mean value ± SD), pmol/L	2.73±1.43	2.73±1.18	0.989
Frequency of abnormal FT3 (%)	0	0	-
FT4 (mean value ± SD), pmol/L	14.85±5.14	13.46±1.64	0.450
Frequency of abnormal FT4 (%)	1/9(11.1%)	0	1
TSH (mean value ± SD), mIU/L	0.27±0.14	1.41±1.47	0.034
Frequency of abnormal TSH (%)	8/9(88.9%)	2/9(22.2%)	0.015
TG-Ab (mean value ± SD), KIU/L	3800.53±10172.02	816.92±2339.95	<0.0001**
Frequency of abnormal TG-Ab (%)	8/9(88.9%)	3/9(33.3%)	0.05
TPO-Ab (mean value ± SD), KIU/L	126.61±107.93	40.06±23.61	0.032**
Frequency of abnormal TPO-Ab (%)	9/9(100%)	6/9(66.7%)	0.206

F, female; M, male; FT4, free thyroxine; FT3, free triiodothyronine; TSH, thyroid-stimulating hormone; TG-Ab, anti-thyroglobulin antibodies; TPO-Ab, antithyroid peroxidase antibody; EDSS: the Expanded Disability Status Scale; ivMP, intravenous methylprednisolone; y, year.

** Mann-Whitney *U*-test.

### Serum TSH, TG-Ab, and TPO-Ab levels were different between patients with and without myelitis

**Figure 2:**
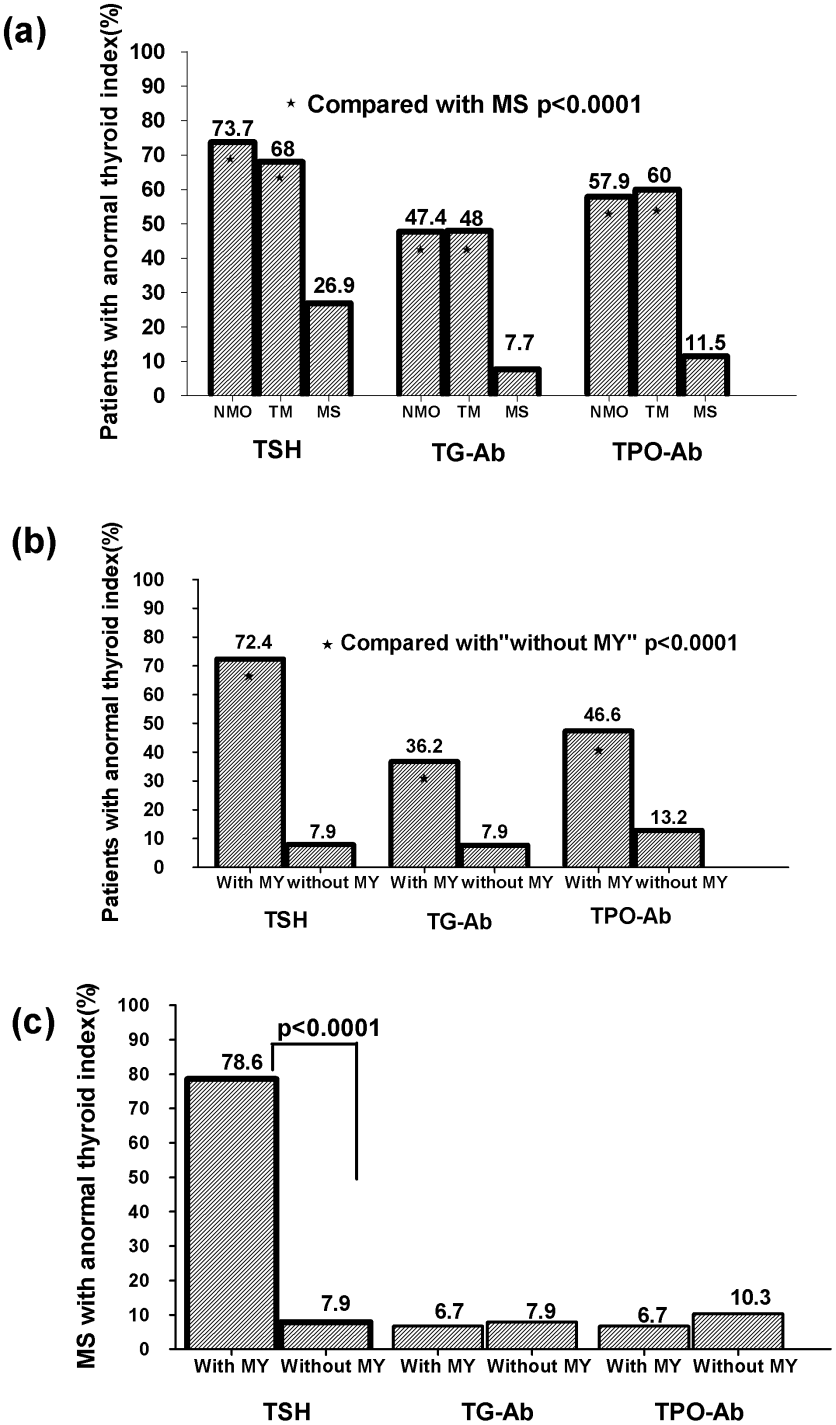
Frequency of abnormal thyroid parameters among NMO, TM, and MS patients at their first attack. (a) In 96 patients, higher numbers of NMO and TM samples had abnormal levels of TSH, TG-Ab and TPO-Ab than MS samples (p<0.0001). (b) The 96 patients were divided into those with (n = 58) and without myelitis (n = 38). TSH, TG-Ab and TPO-Ab levels in patients with myelitis were significantly different to patients without myelitis. (c) Of 52 MS patients, greater numbers with myelitis showed abnormal TSH (p<0.0001) levels compared with those without myelitis.

**Figure 3:**
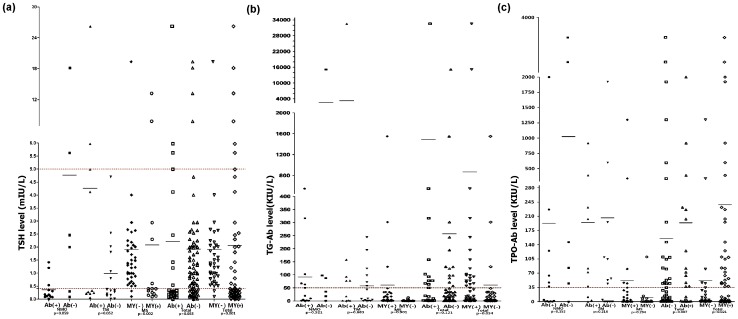
Level of thyroid parameters among different groups at the first attack. (a) In total patients (n = 96) and NMO (n = 19) and TM (n = 48) subgroups, TSH levels were not significantly different between AQP4 antibody positive or negative patients (p>0.05). In total patients (n = 96) and MS subgroup (n = 52), TSH levels were significantly different between patients with and without myelitis (p<0.05). (b) In total patients and of NMO and TM subgroups, TG-Ab levels were not significantly different between AQP4 antibody positive or negative patients (p>0.05). In total patients, TG-Ab levels were significantly different between patients with and without myelitis (p = 0.034). (c) In total patients, AQP4 antibody positive patients had higher TPO-Ab levels than AQP4 antibody negative patients (p = 0.007). Patients with myelitis had higher TPO-Ab levels than patients without myelitis (p = 0.021). Ab: AQP4 antibody; MY: myelitis; +: positive; -: negative; red dashed line: normal range.

Because of the effects of ivMP, we only selected patients who had not received treatment at the first attack. Ninety-six patients were studied (NMO, n = 19; TM, n = 25; MS, n = 52). First, we assessed thyroid parameters among NMO, TM and MS patients. NMO and TM had higher abnormal TSH, TG-Ab and TPO-Ab levels than MS (p<0.0001, Fig 2a). Second, 96 patients were divided into those with myelitis (n = 58) and without myelitis (n = 38). We compared serum levels of FT3, FT4, TSH, TG-Ab, and TPO-Ab at admission (Table 4). Abnormalities of FT3, TSH, TG-Ab, TPO-Ab, gender, and age in patients with myelitis were significantly different to patients without myelitis (Table 4, Fig 2b). Furthermore, levels of TSH, TG-Ab, TPO-Ab in patients with myelitis were significantly different to patients without myelitis (Fig 3a, b, c). According to MRI, myelitis patients could be classified as patients with LETM and patients without LETM. Patients with LETM were not significantly different to patients without LETM, except for abnormal TG-Ab frequency (44.2% *vs*. 14.3%, p = 0.044). Third, 52 MS patients were subdivided into those with myelitis (n = 14) and without myelitis (n = 38). MS patients with myelitis had a higher abnormal TSH frequency (76.8% *vs*. 7.9%, p<0.0001, Figs 2c) and lower median (0.3795 KIU/L *vs*. 1.328 KIU/L, p = 0.026) than MS patients without myelitis.

**Table 4:** Comparison of thyroid parameters in patients with and without myelitis.

Characteristics	Patients with myelitis (n = 58)	Patients without myelitis (n = 38)	p
F/M	44/14	19/19	0.009
Mean age, y (range)**	45.57±14.27 (16–71)	38±13.24(13–67)	0.010
FT3 level (mean value ± SD), pmol/L	3.36±1.34	4.01±0.89	0.010
Frequency of Abnormal FT3 (%)	15/58(25.9%)	2/38(5.3%)	0.010
FT4 level (mean value ± SD), pmol/L	14.55±5.18	15.57±2.92	0.270
Frequency of Abnormal FT4 (%)	3/58(5.2%)	0	0.269
TSH level (mean value ± SD), mIU/L	2.06±4.55	1.91±3.02	0.001*
Frequency of Abnormal TSH (%)	42/58(72.4%)	3/38(7.9%)	<0.0001
TG-Ab level (mean value ± SD), KIU/L	869.83±4675.66	60.73±253.75	0.034*
Frequency of Abnormal TG-Ab (%)	21/58(36.2%)	3/38(7.9%)	0.001
TPO-Ab level (mean value ± SD), KIU/L	238.53±643.60	51.48±215.54	0.021*
Frequency of Abnormal TPO-Ab (%)	27/58(46.6%)	5/38(13.2%)	<0.0001

F, female; M, male; FT4, free thyroxine; FT3, free triiodothyronine; TSH, thyroid-stimulating hormone; TG-Ab, anti-thyroglobulin antibodies; TPO-Ab, antithyroid peroxidase antibody; y, year.

**Mean value ± SD;

*Mann-Whitney *U*-test.

### Serum TSH, TG-Ab, and TPO-Ab levels were different between patients with and without AQP4 antibody

Ninety-six patients without ivMP treatment at the first attack were analyzed (NMO, n = 19; TM, n = 25; MS, n = 52). We assessed thyroid parameters between patients who were AQP4 antibody positive (n = 23) or negative (n = 73). Patients with AQP4 antibody had increased frequencies of abnormal TSH (p = 0.001), TG-Ab (p = 0.004) and TPO-Ab (p<0.0001) than AQP4 antibody negative patients (Table 5). The difference in thyroid parameters between different groups according to AQP4 antibody status is shown in Figs 3a, b, c.

**Table 5:** Comparison of thyroid parameters in 96 patients with and without AQP4 antibodies.

Characteristics	Anti-AQP4(+) (n = 23)	Anti-AQP4(-) (n = 73)	p
F/M	20/3	43/30	0.014
Mean age, y (range)**	47.16±15.40 (17–71)	40.36±13.63(13–69)	0.062
FT3 level (mean value ± SD), pmol/L	5.07±7.58	3.64±1.18	0.133
Frequency of Abnormal FT3 (%)	6/23(26.1%)	11/73(15.1%)	0.228
FT4 level (mean value ± SD), pmol/L	15.58±6.99	14.73±2.99	0.414
Frequency of Abnormal FT4 (%)	2/23(8.7%)	1/73(1.4%)	0.142
TSH level (mean value ± SD), mIU/L	2.22±5.23	1.93±3.43	0.083*
Frequency of Abnormal TSH (%)	17/23(73.9%)	28/73(33.7%)	0.001
TG-Ab level (mean value ± SD), KIU/L	1485.81±6625.76	257.26±1776.4	0.108*
Frequency of Abnormal TG-Ab (%)	11/23(47.8%)	13/73(17.8%)	0.004
TPO-Ab level(mean value ± SD), KIU/L	193.87±442.78	155.23±550.17	0.002*
Frequency of Abnormal TPO-Ab (%)	16/23(69.6%)	16/73(21.9%)	<0.0001

F, female; M, male; FT4, free thyroxine; FT3, free triiodothyronine; TSH, thyroid-stimulating hormone; TG-Ab, anti-thyroglobulin antibodies; TPO-Ab, antithyroid peroxidase antibody; y, year; n, number.

**Mean value ± SD;

*Mann-Whitney *U*-test.

### Independent association between myelitis and thyroid parameters

We analyzed the independent association between myelitis and thyroid parameters in 96 patients by logistic regression analyses. Myelitis was significantly associated with being female (crude OR = 0.318; 95% CI = 0.133–0.763; p = 0.01), age (crude OR = 1.04; 95% CI = 1.008–1.073; p = 0.013), FT3 (crude OR = 6.297; 95% CI = 1.346–29.301; p = 0.019), TSH (crude OR = 30.625; 95% CI = 8.245–113.784; p<0.0001), TG-Ab (crude OR = 6.622; 95% CI = 1.814–24.175; p = 0.004), and TPO-Ab (crude OR = 5.748; 95% CI = 1.966–16.806; p = 0.001). Upon stepwise multiple logistic regression analyses, the independent relationships among TSH (adjusted OR = 33.994; 95% CI = 7.753–149.060; p<0.0001) and TG-Ab (adjusted OR = 7.703; 95% CI = 1.437–41.295; p = 0.017) and myelitis occurrence were confirmed.

Then, we analyzed the risk of myelitis occurrence in 52 MS patients by logistic regression analyses. Myelitis occurrence was associated significantly with TSH (crude OR = 42.778; 95% CI = 7.525–243.184; p<0.0001). Upon stepwise multiple logistic regression analyses, the independent relationships between TSH (adjusted OR = 42.778; 95% CI = 7.525–243.184; p<0.0001) and myelitis occurrence were confirmed.

### A negative AQP4 antibody case with abnormal thyroid parameters

**Figure 4:**
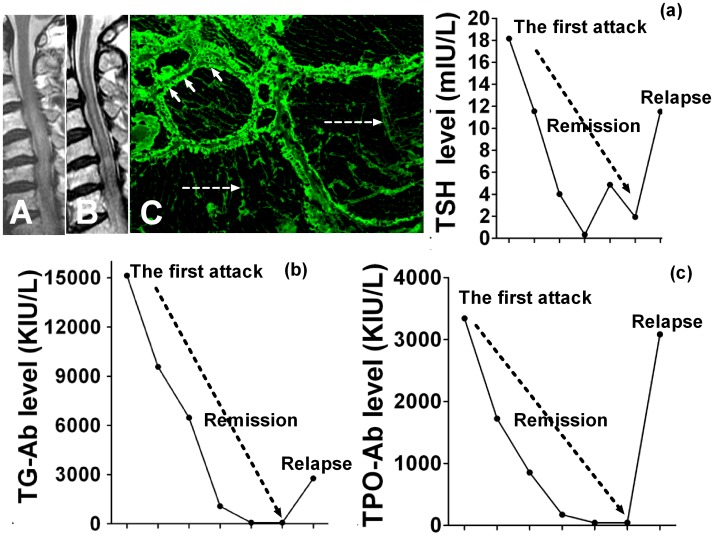
MRI of spinal cord and thyroid parameters of representative cases. A: MRI shows longitudinal extensive lesion in the cervical and thoracic cord (C3-T1) at the first attack; B: At the second attack, T2-weighed spinal MRI shows a lesion extending from C1 to T1; C: Immunofluorescence staining shows serum antibodies bind to the cytoplasmic membranes of follicular cells (short arrow) and thyroid microsoma (dot arrow); (a): Changes of TSH in myelitis attack and remission; (b): Changes of TG-Ab in myelitis attack and remission; (c): (a): Changes of TPO-Ab in myelitis attack and remission.

One female case with age at onset of 64 years had paraplegia and sensory disturbance at the first attack in April 2009. MRI showed a lesion in the cervical and thoracic cord (C3-T1, Fig 4A) and nonspecific lesions in the brain. The serum thyroid function showed abnormally high TSH (18.1463mIU/L), TG-Ab (15144.6 KIU/L) and TPO-Ab (3340.25 KIU/L) levels. After ivMP and intravenous immune globulin treatment, she received oral thyroxine and methylprednisolone. During the remission of 2 years, TSH, TG-Ab and TPO-Ab levels returned to normal levels (Fig 4a, b, c). Two years later, she experienced a second attack with quadriplegia and sensory disturbance. T2-weighed spinal MRI showed a lesion extending from C1 to T1 (Fig 4B). Normal serum TSH, TG-Ab and TPO-Ab levels had increased (Fig 4a, b, c). A cell-based assay showed she was negative for serum AQP4 antibodies (data not shown). However, indirect immunofluorescence showed that high titers (1∶10,000) of sero antibodies could bind to normal monkey thyroid tissues (Fig 4C).

## Discussion

This study demonstrated a relationship between thyroid parameters and demyelinating disease, although there was no evidence to indicate that thyroid disease was involved in the three CNS demyelinating diseases. While MS and NMO are now defined as different entities [2], before the definition of NMO diagnostic criteria in 2006 it is likely that some NMO patients were misdiagnosed as MS, resulting in a less accurate analysis. Misclassification of NMO patients can result in the overestimation of MS prevalence [12]. In the present study, we identified different thyroid parameters among NMO, TM and MS patients at the first attack (Fig 2a), which were present in most patients with NMO or TM. Our results were in accord with a previous study [23], indicating a distinct pattern of thyroid abnormalities in NMOSD and other CNS demyelinating disorders. Previous studies indicated that treatment of MS with interferon and alemtuzumab were associated with the development of other autoimmune disorders [24], [25], [26], [27]. Therefore, results in patients with or without immunomodulatory and immunosuppressive therapy may be different. The present study also identified an interesting effect of ivMP on thyroid parameters. We prospectively studied nine paired patients (NMO, n = 4; LETM, n = 5) during the active stage without treatment before admission. At the active stage, levels of TG-Ab and TPO-Ab were higher in most cases with decreased TSH levels. In that analysis, levels of TG-Ab and TPO-Ab decreased after ivMP administration and in patients whose TSH levels were increased after their EDSS decreased. These observations suggested that thyroid parameters (TSH, TG-Ab, TPO-Ab) could be affected by ivMP. Therefore, although glucocorticoids have no regulatory influence on TSH secretion under physiological conditions [28], [29], we must not neglect the effects of glucocorticoids on thyroid parameters.

Although it is known that some CNS demyelinating disorders predominantly affect the spinal cord, the risk of the occurrence of myelitis is unknown. Thyroid autoimmune diseases have long been observed in optic-spinal MS or TM in Japanese patients [5], [15]. In the present study, we describe, for the first time, a relationship between thyroid parameters and myelitis. First, we observed a higher frequency of abnormal thyroid TSH, TG-Ab and TPO-Ab levels in NMO and TM than in MS patients. Second, we focused on active-stage patients without confounding factors. Patients' with myelitis had greater thyroid parameter abnormalities than patients without myelitis. Furthermore, the length of spinal-cord lesions (LETM or APTM) was not associated with thyroid parameter abnormalities. Our strategy was to analyze the key dependent variables by binary logistic regression analyses. Among several factors, an abnormal level of TSH at baseline was a strong independent factor for myelitis (OR = 33.994). Elevated levels of TG-Ab in serum were observed in 25% (24/96) of the active cohort, and were also an independent factor of myelitis (OR = 7.703). In addition, when MS patients were subdivided into those with or without myelitis, a higher frequency of abnormal TSH was associated with myelitis in MS patients (adjusted OR = 42.778; 95% CI = 7.525–243.184; p<0.0001). Therefore, considering that abnormal levels of TSH or TG-Ab at disease onset are frequently associated with patients with lesions in the spinal cord, it appears that TG-Ab, and especially TSH, might have an as yet unknown role in determining which CNS demyelinating patients have a high risk of developing myelitis.

AQP4 antibodies that selectively target AQP4 have been identified as highly sensitive and specific biomarkers to differentiate NMO from MS. Recent data [23], [30] showed that AQP4 antibody positive patients had more ATAs than AQP4 antibody negative patients and that AQP4 was present in normal follicular cells of the thyroid tissue [31], indicating AQP4 antibody might have a role in thyroid abnormalities. Although we also found that patients with positive AQP4 antibodies had more abnormal thyroid parameters than AQP4 antibody negative patients, the relationship between AQP4 antibody and TSH was not clear. A highly sensitive TSH assay was used in the present study to identify subjects with TSH levels slightly outside the normal range of the assay. These slight abnormalities may be because of transient changes in TSH levels and non-thyroidal illness [27]. We observed fluctuating TSH levels before and after ivMP administration in paired patients. This confirmed that in almost all patients, TSH levels returned to normal in the short term, suggesting transient TSH changes were a response at the acute phase. Furthermore, levels of FT3 and FT4 produced by the thyroid gland [32] remained unchanged in most cases, indicating the lack of local injury in thyroid tissues and a lower sensitivity compared with TSH. A previous study speculated that “molecular mimicry” between thyroglobulin and myelin epitopes should be considered [5]. ATAs can form inflammatory immune complexes and modulate immune responses to myelin basic protein [33], [34]. It was interesting to note the presence of high titers of TG-Ab or TPO-Ab in AQP4 antibody negative patients, indicating the presence of additional target antigens other than AQP4 in thyroid tissues. An AQP4 antibody negative female patient had very high titers of TG-Ab and TPO-Ab, and these fluctuating levels were associated with myelitis relapse. Although there was no evidence that very high ATAs were involved in the pathogenesis of AQP4 negative antibody myelitis, such high ATAs might be involved in the relapsing disease course and exacerbate disability in some patients. Therefore, given the relapsing outcome in AQP4 negative antibody patients with very high ATAs, early immunosuppressive therapy is necessary to attenuate disability. However, a longitudinal study during follow-up should be undertaken to determine whether patients with baseline subclinical thyroid parameters develop clinically significant alterations.

The present study had some limitations. First, because of the retrospective nature of the study, uncontrolled or unknown factors might have affected outcomes and confounded the results. Second, although our description of altered thyroid parameters as a marker for myelitis is interesting, the validation and exploration of the mechanisms whereby these biochemical features influence inflammatory disease of the spinal cord remain to be studied in the future.

In summary, we observed a high prevalence of thyroid parameter abnormalities in patients with NMO and TM than in patients with MS. MS patients with myelitis also had greater TSH levels than MS patients without myelitis. A high prevalence of thyroid parameter abnormalities in patients occurred at relapse, and these abnormalities were affected by ivMP. Using logistic regression analyses, we observed that abnormal TSH and TG-Ab levels were independently associated with myelitis occurrence in patients with CNS demyelinating disorders. Further study of thyroid abnormalities in living CNS demyelinating patients will increase our understanding of disease pathogenesis and special manifestations.
